# Improvement of Attention, Executive Functions, and Processing Speed in Elderly Women as a Result of Involvement in the Nordic Walking Training Program and Vitamin D Supplementation

**DOI:** 10.3390/nu11061311

**Published:** 2019-06-11

**Authors:** Mariusz Lipowski, Tamara Walczak-Kozłowska, Małgorzata Lipowska, Jakub Kortas, Jędrzej Antosiewicz, Giancarlo Falcioni, Ewa Ziemann

**Affiliations:** 1 Department of Health Psychology, Gdansk University of Physical Education and Sport, 80-336 Gdansk, Poland; mariusz.lipowski@awf.gda.pl; 2 Institute of Psychology, University of Gdansk, 80-309 Gdansk, Poland; walczak.tamara@gmail.com; 3 Department of Recreation and Qualify Tourism, Gdansk University of Physical Education and Sport, 80-336 Gdansk, Poland; jakub.kortas@awf.gda.pl; 4 Department of Biochemistry, Gdansk University of Physical Education and Sport, 80-336 Gdansk, Poland; jedrzej.antosiewicz@awf.gda.pl; 5 School of Pharmacy, University of Camerino, 62032 Camerino, Italy; giancarlo.falcioni@unicam.it; 6 Department of Physiology and Pharmacology, Gdansk University of Physical Education and Sport, 80-336 Gdansk, Poland; ewann@awf.gda.pl

**Keywords:** aging, Nordic Walking, nutrition, supplement, performance, cognitive functions

## Abstract

Research indicates that life satisfaction declines with age, and cognitive abilities are gradually reduced—mainly attentional functioning and cognitive processing speed. Therefore, scientists seek to find protective factors and test possible intervention programs; moderately intensive physical activity stands out as particularly promising. In this context, we evaluated the influence of Nordic Walking training supported by vitamin D supplementation (as this nutrient is especially deficient in older people in Poland) on the cognitive and psychological functioning of elderly women. A total of 52 healthy elderly women took part in a Nordic Walking training program complemented by vitamin D supplementation. Cognitive functioning was assessed with the Trail Making Test and the D2 Test of Attention. Quality of life and severity of depressive symptoms were measured with the Short Form Health Survey and the Beck Depression Inventory 2. Significant improvements in all aspects of cognitive functioning was observed (*p* = 0.01–0.47). The study also showed a decrease in depressive symptoms (*p* = 0.026). Physical activity and adequate levels of vitamin D can be the key factors in maintaining self-reliance in old age. Involvement in Nordic Walking training, supported by vitamin D supplementation, can strengthen the cognitive functioning of older people—reflected in higher attentional capabilities, better executive functions, and improved cognitive processing speed.

## 1. Introduction

The widely observed decline in life satisfaction of elderly people is caused by, inter alia, lifestyle changes resulting from retirement [1], negative physical consequences of aging [2], “empty nest” syndrome [3], and gradual depletion of social circles [4]. Old age is often associated with an image of the inevitable limitation of mobility due to loss of muscle mass [4], which, as a result of deterioration, limits everyday functioning, e.g., in dressing, bathing, preparing meals, or climbing stairs [5]. Aging, especially in loneliness, predisposes one toward a depressed mood and the occurrence of depressive symptoms [3,6], the prevalence of which is greater in women [7].

### 1.1. Cognitive Functioning in the Elderly

Cognitive performance declines with age as a result of natural aging processes. The decline is most often observed in domains such as processing speed, reasoning, memory, and executive functions [8]. Certain factors (including lifestyle and personality) may accelerate or slow down the processes associated with these changes (see References [3,9,10]); a sparse social group and loneliness are considered risk factors (e.g., Reference [11]).

Cognitive decline in the elderly is associated with many factors, of which structural changes in the brain and cerebral blood pressure changes are considered central. The decrease in brain volume with aging mainly affects the hippocampus, temporal lobe, and cerebellum [12]. There is also clear evidence of a decrease in white and gray matter [13,14]. Changes are also observed in the concentration of neurotransmitters—more specifically, a decreased density of dopamine receptors, which affects the decrease in effectiveness of attentional mechanisms [15]. Decrease in cerebral blood flow, in particular, may result in reduced attentional capabilities and deterioration of executive functioning due to a higher risk of reduced oxygen supply to the vulnerable prefrontal cortex [16]. Furthermore, physiological factors such as gene expression and hormonal changes also play a significant role in age-related cognitive decline [17,18].

A remedy may be found in involvement in a physical activity which, through the increase of physical fitness, can strengthen self-reliance and contribute to a higher quality of life and social welfare [19,20,21].

### 1.2. Nordic Walking—A Proposition for Physical Activity for the Elderly

Nordic Walking (NW) is a growing trend among middle-aged and elderly people. It involves walking with a pair of poles, similar to those used in skiing, and has its roots in ski training (a “dry run”) outside the winter season. This sport crossed the borders of Scandinavia, becoming popular in other European countries [22]. NW does not require expensive equipment, training can be done almost anywhere on earth, and it seems to be suitable for people with different personality traits and temperaments (including both introverted people and more social people) [20]. Furthermore, this sport is one of the safest; injuries are very rare, which is part of the reason it is recommended for older people [20]. In comparison to normal walking, NW engages additional parts of the body and muscles and, thus, increases the physical benefits of a single training session and diminishes the perceived level of exertion [21]. The main advantage of the use of the Nordic walking poles is the recruitment of the muscles of the upper limbs to produce ground reaction forces resulting in increased energy expenditure [23,24,25]. Nordic walking provides better all-round benefits by improving upper-body strength, cardiovascular endurance, and flexibility than conventional walking [26]. Additionally, NW results in a higher heart rate and higher oxygen consumption then walking [27]. Therefore, the NW is a physical activity recommended for the elderly.

Ossowski et al. [19] indicated an improvement in endurance and increased strength of abdominal muscles, as well as flexibility of the lumbar spinal region, as a result of taking a 15-week program of NW training, held twice a week and lasting 60 min each, with an intensity of 60% of max heart rate. In an earlier study [28], the positive effects of this form of training on strengthening the lower parts of the body were observed as well. Moreover, this type of training significantly increases oxygen consumption [29]; breathing becomes less frequent but is deeper due to increased lung capacity and strength of the respiratory muscles, as well as chest volume and flexibility [21]. Another physiological change is increased blood flow and increased energy supply in people who train with poles in comparison to those who train without poles [30]. Biochemical variables also change in response to systematic NW training; there is a reduction of body mass index, total cholesterol, triglycerides, and low-density lipoproteins, whereas high-density lipoprotein levels tend to increase [31]. The positive effects of moderate to intense training are particularly pronounced in cardiovascular diseases, the incidence of which increases with age [21]. Heart and blood vessels are freed from excessive burden by reduced blood pressure and reduced heart rate. It is important to note that skeletal muscle is a source of myokines, the release of which is induced by exercise. Many myokines may positively influence the functions of other organs, including the heart, adipose tissue, liver, and brain [32]. In addition, the beneficial reduction of total cholesterol and blood thinning is also preventive and remedial. Tschentscher et al. [33] highlighted the beneficial outcomes of physical activity for pulse wave velocity, thereby preventing sclerosis of large vessels (thus, it can be strongly considered to be a protective factor against the development of atherosclerosis). Even shorter training programs appear to be effective, e.g., increased strength and flexibility of the upper and lower parts of the body was observed after a four-week training program [20]. NW is a great choice for people struggling with cardiovascular diseases and patients being treated for cardiosurgical conditions [20,34], as well as in the rehabilitation of people with Parkinson’s disease, as it may contribute to the improvement of spatiotemporal parameters of gait, such as stride length or gait variability [35].

### 1.3. Physical Activity Protecting against Aging Processes

Physical activity is a good remedy for constant tension in muscles, as well as functional dysfunctions in the heart and respiratory organs [21]. Thus, sport is a means of maintaining a state of relaxation of body and mind and contributes to a person’s well-being. The reduction of stress and weight adjustment are the main factors that motivate participation in NW training; however, for older people, it is also a great opportunity to socialize [36]. Involvement in NW training significantly affects levels of health self-assessments [37,38]. Even shorter training periods [20] contribute to a higher self-assessment of the quality of health of women of perimenopausal age. This phenomenon may be partly explained by the positive relationship between the general level of proactive coping strategies, proactive coping, and general quality of life. Skills associated with setting objectives autonomously, using initiative, perseverance in activities, and perceiving events in terms of opportunities for development and self-improvement are important factors contributing to quality of life and successful aging [39]. Moreover, the benefits of NW training are reflected in a decrease of depressive symptoms [40,41], development of coping strategies for anxiety [42], and amelioration of sleeping disorders [43].

Psychogerontologists are looking for modifiable risk factors of reduced cognitive performance in old age. Training basic cognitive processes (working memory and attentional control) appears promising; it improves scores in fluid intelligence assessment [44] and increases sleep duration and physical activity, which strengthens general cognitive functioning [9,45], processing speed and attentional capabilities [46], executive functioning [47], and language and memory [48]. Still, many older people are poorly motivated to undertake physical activity despite the positive effects of training, although subjective physical age seems to be more important for physical activity than chronological age [49].

However, some studies did not find any benefits in cognitively healthy older adults who participated in aerobic physical activities [50] and there are still few reports on the effectiveness of NW training in improving cognitive functioning in the elderly. The most frequently indicated effect is a positive impact on attentional functioning and information processing speed [42]. Yet, there is a lack of data indicating the improvement of executive functions.

Physical activity increases blood flow; thus, muscle fibers receive nutrients and oxygen (which are better absorbed than during the rest period), which results in improved metabolism (faster elimination of metabolic products). Regular exercise contributes to changes in muscle composition; the accumulation of proteins, enzymes, and glycogen enables increased physical activity. Moreover, due to changes in oxygen levels, recovery processes are more efficient [21]. This increase in blood flow obviously goes hand in hand with an increase in cerebral flow, which results in better nourishment and oxygenation of gray and white matter [9,51,52], influencing, for instance, the improvement of the integrity of white matter in the frontal and temporal lobes [53].

### 1.4. Vitamin D Supplementation

It is recognized that many factors can modify the effect of physical activity on improving cognitive functioning. In particular, the concentration of vitamin D is considered to be significant, because vitamin D receptors (VDR) are located in the human cortex and hippocampus, which are key areas for cognition [54]. For instance, Annweiler and colleagues [55], as well as Llewellyn, Lang, Langa, and Melzer [56], found an association between deficiency of 25-hydroxyvitamin D (25-OHD) and cognitive impairments. Pettersen [57] pointed to the improvement in nonverbal (visual) memory due to supplementation of vitamin D in a cohort with insufficient levels of this secosteroid at baseline. The negative effect of low concentration of vitamin D on executive functions (e.g., planning, problem solving, or sequencing) and attention processing speed, but not memory, was indicated by Buell and colleagues [58]. Balion and colleagues [59] indicated a general deterioration of cognitive functions and increased risk of Alzheimer’s disease associated with a deficiency of vitamin D in older people. Additionally, Wicherts and colleagues [60] found a correlation between low concentration of vitamin D (serum 25-OHD below 20 ng/mL) and worse physical functioning, as well as greater decreases in physical performance, in elderly people. These researchers also emphasized the social dimension of the problem of deficiency of vitamin D among older people; more than half of this population has a concentration of serum 25-OHD below 20 ng/mL, which indicates a need for appropriate social strategies focused on spreading the use of supplementing with higher doses in the elderly [60]. Recently published research demonstrates an improvement of cognitive functions in elderly women in response to regular NW training supported by vitamin D supplementation. However, the cognitive response to NW training was not only modified by supplementation, but also by training experience [61]. These obtained results emphasize the importance of regular physical activity.

### 1.5. Present Study

Therefore, how can elderly people benefit from engaging in a Nordic Walking training program supported by vitamin D supplementation? Is the only benefit the physical strengthening of the body? Can it also affect one’s mind and lead to improvements in cognitive functioning, as well as in subjectively perceived quality of life? While the positive physiological and physical changes are characterized quite well in the literature, there are still few accurate reports on changes in the cognitive functioning of older people engaging in NW training supported by vitamin D supplementation. The analysis of previous studies indicates that changes in the scope of attention should be expected. We also assumed that there would be an improvement in executive functions.

Therefore, the aim of this study was to investigate any changes in attention and executive functions, as well as in the perceived quality of life, in elderly women as a result of taking a 12-week NW training course, supported by vitamin D supplementation. Moreover, we decided to investigate whether Nordic Walking training and vitamin D supplementation would reduce the severity of depressive symptoms in this cohort. Such results represent a significant contribution to the discussion about prevention and intervention in mood disorders in the elderly.

We decided to test the following hypotheses:

**Hypothesis** **1.** A 12-week Nordic Walking training program supported by vitamin D supplementation significantly contributes to the improvement of attention and executive functions in older women.

**Hypothesis** **2.** Improvement in quality of life will be observed, especially in the physical and social aspects of functioning, as a result of a 12-week Nordic Walking training program supported by vitamin D supplementation.

**Hypothesis** **3.** The severity of depressive symptoms will decrease due to involvement in a Nordic Walking training program supported by vitamin D supplementation.

**Hypothesis** **4.** The quality of life of older people depends on attentional capabilities, cognitive processing speed, visual–motor coordination, and executive functions, as well as the severity of depressive symptoms.

## 2. Methods

### 2.1. Participants

Fifty-two elderly women (age: mean (M) = 69.78, SD = 4.71) who participate in health promotion programs at the Gdansk University of Physical Education and Sport took part in this study (Figure S1). Before the start of the training program, all persons underwent medical examinations. Exclusion criteria established in the study were uncontrolled hypertension (diastolic blood pressure over 100 mmHg), history of cardiac arrhythmia, cardio-respiratory disorders, and some orthopedic problems. We chose women with the same baseline vitamin D3 concentration (above 20 ng/mL) in order to ensure that the observed changes would not be affected by vitamin D deficiency. Many women in Poland practice NW training regularly (it is often on offer in local cultural centers, among other places). Thus, we chose women with different levels of physical training experience: beginner and advanced (more than four years of regular NW training) participants. Therefore, 35 women qualified for the advanced group and the remaining 17 women constituted the beginner group (supplement).

### 2.2. Procedure

#### 2.2.1. Training Sessions

The Nordic Walking training program lasted 12 weeks, during which 35 training units, divided into three micro-cycles, took place. Each training session started in the morning and all participants followed the same routine. They ate a light breakfast and trained 1 h later; the training lasted 1 h (10-min warm-up, about 40 min of Nordic Walking, and 10-min cool-down) and had a 60–70% intensity of maximal heart rate (HR). Training sessions were held three days a week (Monday, Wednesday, and Friday) and, through the whole experiment, the same group of research assistants and coaches supervised all trainings.

#### 2.2.2. Vitamin D Supplementation

Blood samples were collected in order to assess levels of vitamin D3 concentration before 12 weeks of Nordic Walking training and were taken by a professional nurse between 7:00 and 8:00 a.m. following an overnight fast. The blood was taken from the antecubital vein into a vacutainer. Determination of the concentration of 25-hydroxy D3 (25OHD3), a vitamin D metabolite, was carried out on the basis of the ultra-fast LC/MS/MS analysis of 25-OH Vitamin D2 and D3 from serum, with modifications, using the Phenomenex TN-1055 application. A mass spectrometer (SHIMADZU LCMS 8050 HPLC system, Nexera X2 column, Agilent Eclipse Plus C18 1.8 μm, 2.1 × 100 mm) was used for the assay [61]. During the Nordic Walking training program, the participants’ diets were complemented with vitamin D supplementation (4000 IU/day) in order to ensure that the observed changes would not be affected by vitamin D deficiency (common among people in Poland). Furthermore, participants of the study were asked not to change their eating habits during the program.

#### 2.2.3. Cognitive and Psychological Assessment

The assessment of cognitive skills and psychological functioning was done twice: before and after the training period. The interval between both tests was 12 weeks. Each participant was examined individually by the same trained researcher–psychologist.

#### 2.2.4. Ethics Statement

The study received the official approval of the Bioethical Committee of the Regional Medical Society in Gdansk (KB-34/18), in accordance with the Declaration of Helsinki. All participants were given detailed information about the experiment and gave written consent to participate before the start of the experiment. The purpose of obtaining the participants’ consent was so that referrals could be made to their physicians to exclude possible pathologies, as well as for submitting medical details to review by the study’s medical officer.

### 2.3. Measurement Tools

#### 2.3.1. NW Training Units

The Garmin Forerunner 405 with built-in global positioning system (GPS) was used for recording each training unit. Additionally, each participant received a sport-tester type device used for cardiovascular control once a week. A group consisting of the same research assistants and coaches supervised all training sessions. Standard poles with special NW gloves but no additional telescopes were used for training. Participants did not take part in any other types of exercise throughout the duration of the study; they were also instructed not to change their daily habits (for example, daily activity with a grandchild). Training attendance was checked by the instructor; it averaged within the range of 85–90%.

#### 2.3.2. D2 Test of Attention (D2)

The D2 test allows the assessment of selectivity and concentration of attention, as well as the visual ability to search images [62]. This paper–pencil test is used to examine children and adults during individual meetings or in a group session. It takes a total of about 10 min to complete, and the participant’s task is to strike the letters d with two dashes: at the top, bottom, or one on the top and one at the bottom (”d /„d /,d’) and ignore all other stimuli. These letters are listed in 14 sequences of the letters p and d (each row consisting of 47 stimuli), varying in the number of bars (1–2) above and below the letter. The participant has 20 s to cross out reference letters on a single row after which they proceed to the next one without pausing. The test allows the calculation of the following indicators after completion: total number of stimuli processed (TN), which indicates information processing speed; percentage errors (%E); the total number of errors (TN-E; number of stimuli not crossed out and the number of stimuli incorrectly crossed out), which reflects general perception ability; and the concentration performance indicator (CP), which is the difference between correctly and incorrectly delineated stimuli.

#### 2.3.3. Trail Making Test A&B (TMT A&B)

The TMT is a part of the Halstead–Reitan Battery (Polish version by Kądzielawa [63]). This clinical tool is used to assess cognitive processing speed and visual–motor coordination (TMT-A), as well as visuo-spatial working memory and executive functions (TMT-B). The TMT consists of two parts—A and B. In the first one (A), the task of the participant is to connect the numbers 1–25 as fast as possible in numerical order with a continuous line. In part B, the participant has to connect the numbers with consecutive letters of the alphabet as fast as possible in an alternating way according to the formula: 1–A–2–B–3–C–4–D, etc. The final result in both parts is the time measured in seconds obtained in part A and part B [64].

#### 2.3.4. Short Form Health Survey (SF-36 v. II)

The SF-36 v. II is a paper–pencil questionnaire which is used to assess the quality of life in relation to one’s health. This tool consists of eight subscales measuring both physical and mental aspects of health: vitality, bodily pain, general health, physical functioning, social functioning, physical role functioning, emotional role functioning, and mental health. For the purpose of this study, SF-36 was used to detect changes in the subjectively perceived health and well-being of participants [65].

#### 2.3.5. The Beck Depression Inventory (BDI-2)

The BDI-2 was used to assess the severity of depressive symptoms in participants. It consists of 21 items scored from zero to three points, thus giving the possibility of a total from 0 to 63 points. Higher scores on the BDI-2 correspond to a higher level of depression. The interpretation of results includes four categories: no depression (0–9), mild depression (10–16), moderate depression (17–29), and severe depression (30–63) [66].

#### 2.3.6. Statistical Analysis

Statistical analyses were performed using SPSS v. 24 for Windows, licensed to the University of Gdansk. After analyzing their distributions with the Shapiro–Wilk test, descriptive characteristics of variables were expressed using means and standard deviations (SD). In order to verify the hypotheses, a series of Student’s *t*-tests for dependent variables, Pearson’s correlations, and regression analyses were carried out. Post–pre-exercise changes in attention and executive functions (hypothesis 1), quality of life (hypothesis 2), and depressive symptoms (hypothesis 3) were analyzed using a *t*-test for repeated measures. Pearson’s correlations and regression analyses were carried out to investigate hypothesis 4, regarding the influence quality of life on attentional capabilities, cognitive processing speed and visual–motor coordination, executive functions, and severity of depressive symptoms. Additionally, an ANOVA for repeated measurements was used to assess the differences in these variables depending on training experience. The *p*-values obtained as less than 0.01 were considered statistically significant.

The sample size was predetermined using power calculation. The estimated values of mean and SD from preliminary tests with 20 exercising women predetermined the sample size of the exercising group used in tests with power 0.8 to be *n* participants.

The statistical power of the study was calculated after the study using a post hoc statistical power calculator for the Student’s *t*-test.

## 3. Results

### 3.1. Nordic Walking with Vitamin D Supplementation and Cognitive Functioning Improvement

The 12 weeks of Nordic Walking training supported by vitamin D supplementation induced significant changes in all four indicators of attentional functioning assessed by the D2 Test of Attention. Improvement in information processing speed was observed in a higher number of stimuli processed (*t* = −1.64, *p* = 0.054, M = 405.39 on the first assessment vs. M = 419.91 on the second assessment). A decrease in the percentage of errors reflected an increase in accuracy (*t* = 3.36, *p* = 0.001, M = 9.39% on the first assessment vs. M = 7.71% on the second assessment). General perception ability also improved as a result of the NW training program, which was reflected in the higher value of total number of stimuli processed minus errors in the second measurement (*t* = −2.37, *p* = 0.011, M = 364.90 on the first assessment vs. M = 383.84 on the second assessment). Additionally, a significant change was observed in concentration performance, indicated by the greater difference between correctly and incorrectly delineated stimuli in the second measurement (*t* = −3.10, *p* = 0.002, M = 132.96 on the first assessment vs. M = 142.53 on the second assessment). The details of these analyses are presented in Table 1.

Additionally, we observed a significant interaction, F (1, 50) = 4.79, *p* < 0.05, ƞ^2^*p* = 0.09, between the change in the accuracy (percentage errors) and training experience (beginners and advanced). The percentage errors at baseline was lower and the decrease was greater in the advanced group (M = 9.04% on the first assessment vs. M = 6.62% on the second assessment) in comparison to beginners (M = 10.11% on the first assessment vs. M = 9.95% on the second assessment).

The Nordic Walking training program supported by vitamin D supplementation also led to an improvement in cognitive processing speed and visual–motor coordination, reflected in shorter times on part A of the TMT at the second measurement (*t* = 1.71, *p* = 0.047, M = 30.14 s at the first assessment vs. M = 27.93 s at the second assessment). Positive changes, as we expected, were also observed in executive functions as a result of the training (*t* = 1.76, *p* = 0.043, M = 87.17 s on the first assessment vs. M = 79.07 s on the second assessment). Results of the TMT are presented in Table 2.

Thus, the first hypothesis, regarding the influence of the 12-week Nordic Walking training program supported with vitamin D supplementation on improvement of attention and executive functions of older women, was confirmed in our study.

### 3.2. Nordic Walking with Vitamin D Supplementation and Quality of Life Improvement

The 12 weeks of Nordic Walking training supported by vitamin D supplementation caused a significant change (decrease) in one aspect of subjectively perceived quality of life—general health (*t* = 2.48, *p* = 0.009, M = 51.44 s on the first assessment vs. M = 47.02 s on the second assessment). We observed a lack of significant differences between the first and second measurements in the other components of the quality of life (Table 3).

In view of the above, it should be recognized that the second hypothesis, regarding the influence of the 12 weeks of Nordic Walking training supported with vitamin D supplementation on the improvement in the quality of life, was only partially confirmed.

### 3.3. Severity of Depressive Symptoms after Nordic Walking Program and Vitamin D Supplementation

Improvement in mental functioning was observed in the decrease of severity of depressive symptoms (*t* = 2.00, *p* = 0.026, M = 8.83 s on the first assessment vs. M = 7.73 s on the second assessment). Details are presented in Table 4.

Therefore, we should accept the third hypothesis about the reduction of depressive symptoms as a result of involvement in the NW training program.

### 3.4. Quality of Life, Cognitive Functioning, and Severity of Depressive Symptoms

A series of regression analyses were carried out and we identified the predictors of the quality of life of people participating in the program before and after the participation. The explanatory variables in the analysis were processing speed (TN), accuracy (%E), general perception ability (TN-E), concentration (CP), cognitive processing speed and visual–motor coordination (TMT-A), executive functions (TMT-B), and severity of depressive symptoms (BDI-2), and the variables explained were the various aspects of the quality of life.

#### 3.4.1. Before Participating in the Program

**Figure 1:**
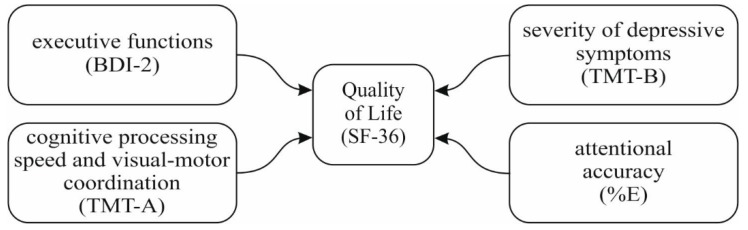
Predictors of the quality of life of women participating in the 12-week Nordic Walking training program supported by vitamin D supplementation.

In the initial stage, due to the problem of collinearity, we had to remove the following variables from the analysis: TN, TN-E, and CP. The final model was as shown in Figure 1.

For the first aspect of quality of life—physical functioning—only the severity of depression symptoms (β = −0.38, standard error (SE) = 0.37, *t* = −2.63, *p* < 0.05) turned out to be a significant predictor of quality of life (summary of the model: *R*^2^ = 0.15, F(4, 51) = 2.03, *p* = 0.105). The same situation existed for role limitations due to physical health (summary of the model: *R*^2^ = 0.15, F(4, 51) = 2.00, *p* = 0.110; severity of depression symptoms: β = −0.38, SE = 0.44, *t* = −2.63, *p* < 0.05). In the case of bodily pain (summary of the model: *R*^2^ = 0.17, F(4, 51) = 2.36, *p* = 0.067), the only significant predictor was executive functions (β = 0.33, SE = 0.05, *t* = 2.10, *p* < 0.05). For general health (summary of the model: *R*^2^ = 0.25, F(4, 51) = 3.97, *p* < 0.01), the sole significant predictor was the severity of depression symptoms (β = −0.54, SE = 0.38, *t* = −3.98, *p* < 0.01). Results were analogous for vitality (summary of the model: *R*^2^ = 0.20, F(4, 51) = 2.91, *p* < 0.05; severity of depression symptoms: β = −0.41, SE = 0.39, *t* = −2.94, *p* < 0.01), social functioning (summary of the model: *R*^2^ = 0.32, F(4, 51) = 5.43, *p* < 0.01; severity of depression symptoms: β = −0.52, SE = 0.44, *t* = −4.02, *p* < 0.01), role limitations due to emotional problems (summary of the model: *R*^2^ = 0.22, F(4, 51) = 3.38, *p* < 0.05; severity of depression symptoms: β = −0.41, SE = 0.45, *t* = −2.96, *p* < 0.01), mental health (summary of the model: *R*^2^ = 0.31, F(4, 51) = 5.22, *p* < 0.01; severity of depression symptoms: β = −0.47, SE = 0.37, *t* = −3.59, *p* < 0.01), physical health component (summary of the model: *R*^2^ = 0.27, F(4, 51) = 4.44, *p* < 0.01; severity of depression symptoms: β = −0.54, SE = 0.27, *t* = −4.06, *p* < 0.01), mental health component (summary of the model: *R*^2^ = 0.37, F(4, 51) = 6.96, *p* < 0.01; severity of depression symptoms: β = −0.55, SE = 0.30, *t* = −4.43, *p* < 0.01), and quality of life index (summary of the model: *R*^2^ = 0.37, F(4, 51) = 6.89, *p* < 0.01; severity of depression symptoms: β = −0.59, SE = 0.25, *t* = −4.79, *p* < 0.01).

#### 3.4.2. After the Program

In the case of all regression analyses for the second measurements, as with the first measurements, we had to remove the following variables from the analysis due to collinearity: TN, TN-E, and CP. The final model was as previously presented (Figure 1). The regression analysis with physical functioning as dependent variable revealed that only the severity of depression symptoms (β = −0.42, SE = 0.35, *t* = −3.22, *p* < 0.01) was a significant predictor of the quality of life in this aspect (summary of the model: *R*^2^ = 0.20, F(4, 51) = 2.10, *p* < 0.05). As for the second aspect of the quality of life, role limitations due to physical health (summary of the model: *R*^2^ = 0.31, F(4, 51) = 5.18, *p* < 0.01), like before, the severity of depression symptoms (β = −0.40, SE = 0.44, *t* = −3.26, *p* < 0.01) and also executive functions (β = −0.35, SE = 0.08, *t* = −2.76, *p* < 0.01) constituted important predictors. In the case of bodily pain (summary of the model: *R*^2^ = 0.25, F(4, 51) = 3.99, *p* < 0.01), the only significant predictor was the severity of depression symptoms (β = −0.49, SE = 0.23, *t* = −3.83, *p* < 0.01). Similarly, for general health (summary of the model: *R*^2^ = 0.33, F(4, 51) = 5.66, *p* < 0.01), the sole significant predictor was the severity of depression symptoms (β = −0.56, SE = 0.29, *t* = −4.64, *p* < 0.01). The same situation occurred for vitality (summary of the model: *R*^2^ = 0.39, F(4, 51) = 7.34, *p* < 0.01; severity of depression symptoms: β = −0.61, SE = 0.33, *t* = −5.27, *p* < 0.01) and social functioning (summary of the model: *R*^2^ = 0.39, F(4, 51) = 7.60, *p* < 0.01; severity of depression symptoms: β = −0.45, SE = 0.33, *t* = −4.84, *p* < 0.01). In the case of role limitations due to emotional problems (summary of the model: *R*^2^ = 0.32, F(4, 51) = 5.43, *p* < 0.01) the severity of depression symptoms (β = −0.43, SE = 0.44, *t* = −3.54, *p* < 0.01) and also executive functions (β = −0.33, SE = 0.08, *t* = −2.68, *p* < 0.01) constituted important predictors of quality of life in this aspect. As for mental health (summary of the model: *R*^2^ = 0.49, F(4, 51) = 11.33, *p* < 0.01) the severity of depression symptoms (β = −0.66, SE = 0.25, *t* = −6.26, *p* < 0.01), as well as cognitive processing speed and visual–motor coordination (β = −0.25, SE = 0.15, *t* = −2.33, *p* < 0.05) constituted important predictors of quality of life in this aspect. In the case of the physical health component (summary of the model: *R*^2^ = 0.39, F(4, 51) = 7.61, *p* < 0.01), the only significant predictor was the severity of depression symptoms (β = −0.57, SE = 0.24, *t* = −4.99, *p* < 0.01). For the mental health component (summary of the model: *R*^2^ = 0.56, F(4, 51) = 14.65, *p* < 0.01), it was the severity of depression symptoms (β = −0.68, SE = 0.24, *t* = −7.03, *p* < 0.01), as well as the cognitive processing speed and visual–motor coordination (β = −0.21, SE = 0.14, *t* = −2.10, *p* < 0.05) that constituted important predictors of quality of life in this aspect. In the case of the quality of life index (summary of the model: *R*^2^ = 0.53, F(4, 51) = 13.23, *p* < 0.01), the severity of depression symptoms (β = −0.68, SE = 0.21, *t* = −6.78, *p* < 0.01), as well as executive functions (β = −0.22, SE = 0.04, *t* = −2.11, *p* < 0.05) constituted important predictors of quality of life (in general).

Regarding the results obtained in regression analyses, it should be concluded that the fourth hypothesis, about the quality of life of older people depending on their attentional capabilities, cognitive processing speed, visual–motor coordination, executive functions, and severity of depressive symptoms, was only partially confirmed.

#### 3.4.3. Changes in Vitamin D Concentration

The baseline concentration of vitamin D was above the recommended range and was equal to 24.93 ± 10.35 ng/mL. The applied procedure induced an increase in vitamin D among our group of seniors. Post-intervention, vitamin D significantly increased to 57.36 ± 39.10 ng/mL. Nonetheless, values of vitamin D did not correlate with cognitive functions.

## 4. Discussion

It seems that, for the optimization of functioning in the elderly, physical activity and proper nutrition are key factors in maintaining health. As our own study shows, a 12-week training program supported by vitamin D supplementation improved the cognitive functioning of elderly participants. The change was observed in information processing speed, general perception ability, concentration, visual–motor coordination, and executive functions (EF). The beneficial effect of physical activity that we found was also confirmed by other studies, as mentioned in the introduction (e.g., References [46,47]). Our research enhances the data beyond what is known; it indicates the influence of NW training sessions with vitamin D supplementation on other psychological variables (severity of depression symptoms and quality of life) in elderly women and, in addition, it points to some interrelations between the quality of life and cognitive and psychological indicators.

### 4.1. Attention and Executive Functions

The study confirmed our initial assumption, based on previous theoretical considerations, that moderate to intense training, in the form of Nordic Walking, contributes to improvement in cognitive functioning. More precisely, it contributes to improvement in attentional functioning manifested in concentration performance, as well as increases in accuracy during the continuous performance task. We also observed a positive change in general perception ability and processing speed on the same task, but in terms of different indicators. It should be noted that, despite the positive impact of training on the parameters of attention (measured with the D2 Test of Attention), in both the first and the second measurement, they were in the normal range (the results were compared to the norms for the population aged 18–59.11 [67]). Thus, it can be concluded that this form of training not only positively influences people with reduced cognitive abilities, but also strengthens normal cognitive abilities. Additionally, the performance on the first part of the Trail Making Test confirmed the improvement in cognitive processing speed, as well as visual–motor coordination. The average result obtained in TMT-A by participants in the second measurement was close to (even slightly lower than) the average score in a population of people aged 18–68 (M = 28.11, SD = 10.84) [68].

In this study, executive functions also seemed to improve due to NW training supported by vitamin D supplementation. We observed positive changes in cognitive flexibility, which is one aspect of executive functioning, alongside working memory and inhibition in Miyake et al.’s three-dimensional EF theory [69]. Still, the results obtained on the first and second measurements were higher (longer time in seconds) than the average score in a population of people aged 18–68 (M = 51.38, SD = 16.53) [68]. This clearly indicates the high sensitivity of executive functions to the aging process; however, at the same time, it shows that EF can be improved in the elderly by engaging in physical activity and optimal diet. The frontal lobes, strongly associated with executive functioning, are particularly vulnerable to damage and reduced effectiveness with age. Therefore, moderate training (e.g., NW) combined with vitamin D supplementation can be a protective factor for functions related to this area of the brain.

### 4.2. Quality of Life

On the basis of a thorough analysis of the literature on the subject, we assumed that participation in the training program would contribute to the improvement of the indicators of quality of life in older women. Yet, an improvement in quality of life was not observed in this study. We suppose that this observation may be due to the fact that the participants were healthy older people, who did not suffer from any diseases, and the SF-36 measures quality of life in relation to one’s health. Perhaps if we had used another tool to measure quality of life, we would have found differences. Previous research indicated increases in several aspects of quality of life as a result of NW training supported by vitamin D supplementation [20,21]; therefore, when planning further research, it is worth considering using another questionnaire. Perhaps given a longer period of regular training or a higher dosage of vitamin D we could observe changes in the group, as they may begin to feel greater satisfaction resulting from physical changes, such as strengthening of the body and muscles or increased endurance.

### 4.3. Depressive Symptoms

In this study, we also assumed that there would be an improvement in the indicators concerning the mental state of women participating in the study. In contrast to the results regarding quality of life in our experiment, NW activity and vitamin D supplementation influenced women’s mental state—reflected in a decrease of depressive symptoms. Similar observations were found in previous research [40,43], indicating that this type of training can be an important preventive factor, as well as be useful in developing intervention programs for mood disorders in the elderly. As indicated in the introduction, older people may more frequently experience decreased mood and manifest symptoms of depression. However, it should be noted that the results of the surveyed women (both before and after the program) were within the normal range (with reference to the interpretation proposed by Beck [66]). Therefore, it seems an interesting idea to run an experiment on a group of women diagnosed with depression or other mood disorders, to see if their indicators would also improve.

### 4.4. Quality of Life Predictors

In this study, we also looked for predictive factors for quality of life in the older women. Cognitive processing speed and visual–motor coordination turned out to be predictors of mental aspects of quality of life (mental health and mental health component) in the second measurement (after the program). Additionally, our analyses indicated that executive functions turned out to be a significant predictor of several aspects of quality of life. In the first assessment (before participating in the program), EF predicted only bodily pain. However, in the second assessment (after the program), EF predicted role limitations due to physical health, role limitations due to emotional problems, and also quality of life index. We can conclude that changes in executive functions, as a result of participating in the NW training program with vitamin D supplementation, are reflected in a sense of being able to fulfil the life roles on a physical and emotional level.

Additionally, we found that the severity of depressive symptoms is a more important predictor of the quality of life (in almost every aspect) than cognitive functioning. Thus, we concluded that the quality of life of elderly women depends strongly on their mental state and, if these depressive symptoms are reduced as a result of involvement in NW training and vitamin D supplementation, there is a sense that these would increase the quality of life in the long term. As mentioned in the introduction, proactive coping strategies and proactive coping reflect an improved quality of life [39]. When we observe deficits in skills connected with setting objectives autonomously, using initiative, perseverance in activities, and perceiving events in terms of opportunities for development and self-improvement—i.e., aspects associated with depressive symptoms—people (especially older people) may experience less satisfaction from their own life.

While access to vitamin D supplementation is generally available and relatively easy to incorporate into one’s daily diet, finding the motivation for regular physical activity seems to be much more difficult. Still, despite many benefits and more spare time, elderly people dedicate a small proportion of time to sports and recreation [70,71]. Older people are advised to get about 150 min of moderate to vigorous activity per week; however, only a small percentage of them meet these requirements [71]. The lack of regular physical activity in the elderly is most often the result of bad habits formed in their years of highest productivity, where the nature of their work often minimized their activity, and additional family responsibilities reduced the time allocated to sport and recreation (e.g., Reference [20]). Moreover, sports centers promote youth and attractiveness, making it difficult for older people to fit in [20]. Thus, NW seems to be a good year-round alternative for people who do not feel comfortable in sports centers. However, if we expect a positive effect from training, it must be regular and intense enough (it must involve expending enough energy). Of course, to maintain commitment to training and to have a positive effect on the mood, it should also be pleasant and give a sense of a mental comfort and security. Moreover, Scicchitano and colleagues [72] indicated that nutraceuticals and functional food ingredients that are beneficial to vascular health may represent useful compounds that are able to reduce overall cardiovascular risk induced by dyslipidemia, by acting parallel to statins, or as adjuvants in case of failure or when statins cannot be used.

Therefore, the challenge is to increase the motivation of older people to begin the initial training, as positive changes, after only a few weeks, can be expected to be helpful in sustaining this commitment.

## 5. Limitations

We consider the small group of participants and the lack of assessment of physical capacity to be limitations of the study. In order to avoid physical and mental fatigue before the training program, we tried to schedule and complete each task on separate days. We did not want to discourage participants and lower their enthusiasm by causing them to feel physically fatigued before the start of the program. The smaller number of the group performing the first training is related to the need for greater control of this group during the exercise.

Using questionnaire methods to assess occurrence of depressive symptoms and level of quality of life might also be seen as a limitation because they are based on self-report and not on objective diagnostics. We decided to use BDI-II and SF-36 II because subjective assessment of one’s well-being was the most important for our research.

## 6. Conclusions

1. The involvement in Nordic Walking training supported by vitamin D supplementation can strengthen the cognitive functioning of older people—specifically, attention and executive functions.
2. Nordic Walking training with vitamin D supplementation may have a partial influence on the perceived quality of life of older women.
3. Nordic Walking training can contribute to the improvement in the mental health of older women, reflected in a decrease of depressive symptoms.
4. Improvement of cognitive functions and mental state, as a result of involvement in the Nordic Walking training, may partially transfer to subjectively perceived quality of life.
5. Physical activity and proper nutrition can be key factors in maintaining self-reliance in old age.

## Figures and Tables

**Table 1:** The results of selective attention (D2) before and after 12 weeks of the Nordic Walking training program.

Dependent Variables	First Measurement		Second Measurement		*t*-Test	*p*-Value	Post Hoc Power
	M	SD	M	SD			
TN ^1^	405.39	88.39	419.91	93.85	−1.64	n.s.	0.16
%E ^2^	9.39%	4.60%	7.71%	5.26%	3.36	0.001	0.40
TN-E ^3^	364.90	82.34	383.84	82.78	−2.37	0.011	0.26
CP ^4^	132.96	33.33	142.53	32.09	−3.10	0.002	0.34

M—mean; n.s.—not significant. ^1^ TN—total number of stimuli processed; ^2^ %E—percentage errors; ^3^ TN-E—general perception ability; ^4^ CP—concentration performance indicator.

**Table 2:** The results of cognitive processing speed (Trail Making Test (TMT)-A) and executive functions (TMT-B) before and after 12 weeks of the Nordic Walking training program.

Dependent Variables	First Measurement		Second Measurement		*t*-Test	*p*-Value	Post Hoc Power
	M	SD	M	SD			
TMT-A ^1^ (s)	30.14	6.74	27.93	9.39	1.71	0.047	0.28
TMT-B ^2^ (s)	87.17	27.69	79.07	31.45	1.76	0.043	0.32

^1^ TMT-A—cognitive processing speed and visual-motor coordination; ^2^ TMT-B—visuo-spatial working memory and executive functions.

**Table 3:** The results of physical and mental aspects of health (Short Form Health Survey (SF-36)) before and after 12 weeks of the Nordic Walking training program and vitamin D supplementation.

Dependent Variables	First Measurement		Second Measurement		*t*-Test	*p*-Value	Post Hoc Power
	M	SD	M	SD			
Physical functioning	82.70	13.98	81.17	14.96	0.80	n.s.	0.08
Role limitations due to physical health	68.75	16.79	69.02	19.84	−0.11	n.s.	0.06
Bodily pain	20.42	9.32	18.30	9.77	1.34	n.s.	0.22
General health	51.44	15.11	47.02	13.01	2.48	0.009	0.38
Vitality	68.50	15.06	69.28	15.65	−0.40	n.s.	0.08
Social functioning	82.25	18.70	78.46	21.39	1.33	n.s.	0.20
Role limitations due to emotional problems	82.17	17.70	79.79	19.92	1.13	n.s.	0.14
Mental health	75.10	15.35	77.34	13.09	−1.14	n.s.	0.16
Physical health component	60.41	10.93	58.35	11.74	1.55	n.s.	0.18
Mental health component	75.75	13.23	75.72	13.52	0.02	n.s.	0.06
Quality of life index	67.45	10.99	66.32	11.57	0.88	n.s.	0.10

**Table 4:** The results of severity of depressive symptoms (Beck Depression Inventory (BDI-2)) before and after 12 weeks of the Nordic Walking training program.

Dependent Variables	First Measurement		Second Measurement		*t*-Test	*p*-Value	Post Hoc Power
	M	SD	M	SD			
BDI-2	8.83	5.46	7.73	5.50	2.00	0.026	0.18

## Supplementary Materials

The following are available online at https://www.mdpi.com/2072-6643/11/6/1311/s1: Figure S1: Study flow diagram of the progress through the phases of experiment.
