# Supplementary figures and images for: Improvement of Attention, Executive Functions, and Processing Speed in Elderly Women as a Result of Involvement in the Nordic Walking Training Program and Vitamin D Supplementation
Source: Nutrients. 2019 Jun 11;11(6):1311. doi: 10.3390/nu11061311 (PMC6628124; doi:10.3390/nu11061311)

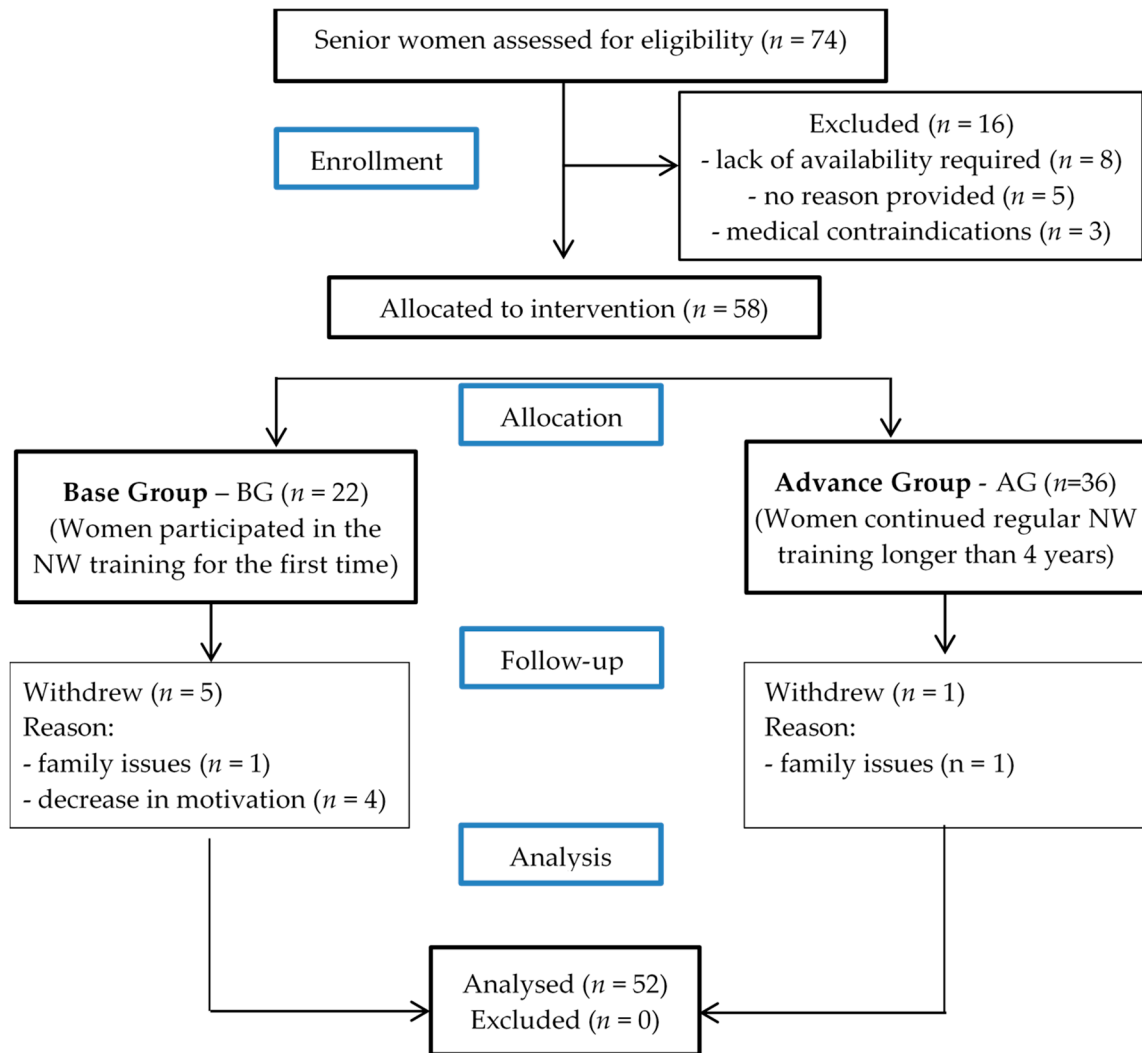

**Figure S1.** Study flow diagram of the progress through the phases of experiment.

Supplement: Supplementary file 1 [file nutrients-11-01311-s001.pdf]
